# Supplementary material for: Phytochemical Investigation of Lepionurus sylvestris Blume and Their Anti-Diabetes Effects via Anti-Alpha Glucosidase and Insulin Secretagogue Activities Plus Molecular Docking
Source: Pharmaceuticals (Basel). 2023 Aug 10;16(8):1132. doi: 10.3390/ph16081132 (PMC10458858; doi:10.3390/ph16081132)
Supplement: Supplementary file 1 [file pharmaceuticals-16-01132-s001.zip › pharmaceuticals-2476520-supplementary.pdf]

Supplementary Materials

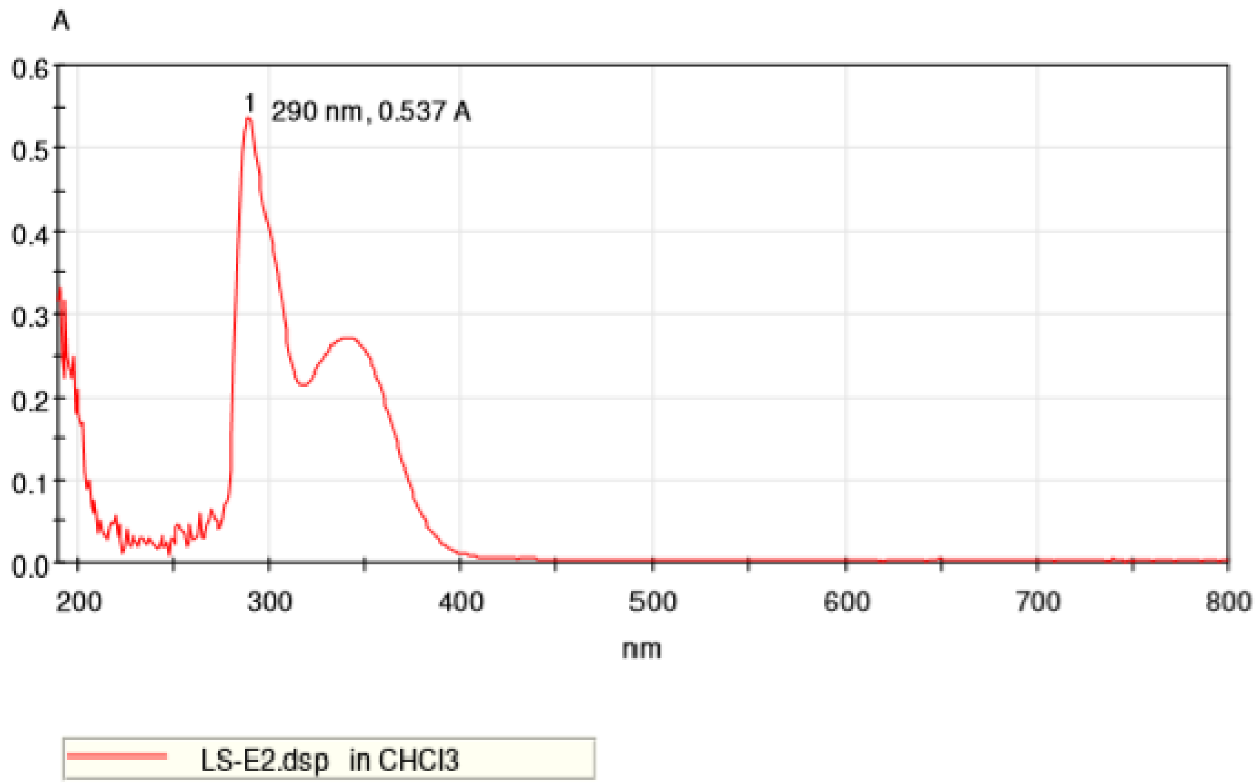

Figure S1. UV-Visible spectrum of interruptin A in chloroform

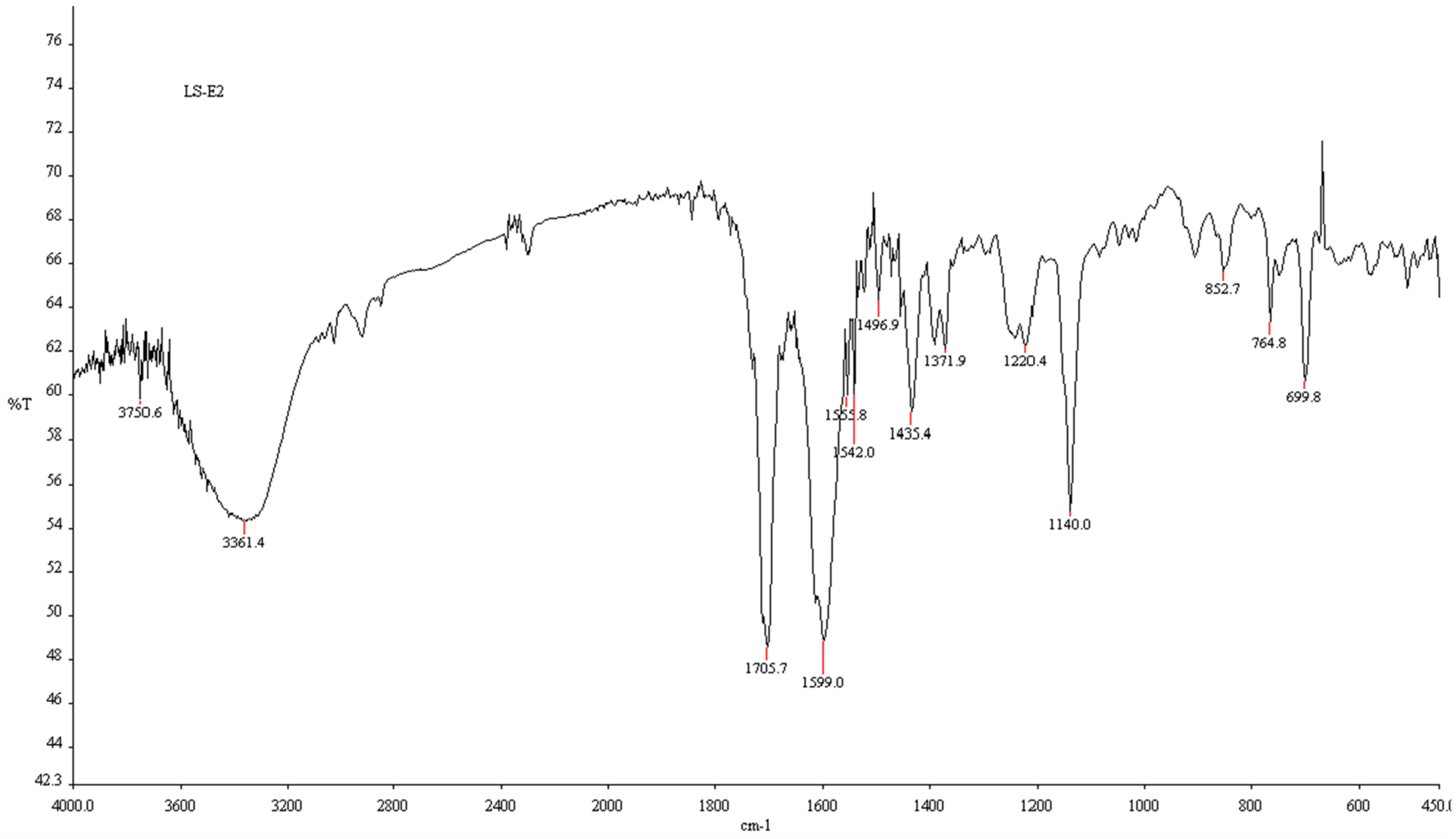

Figure S2. IR spectrum of interruptin A in KBr disc

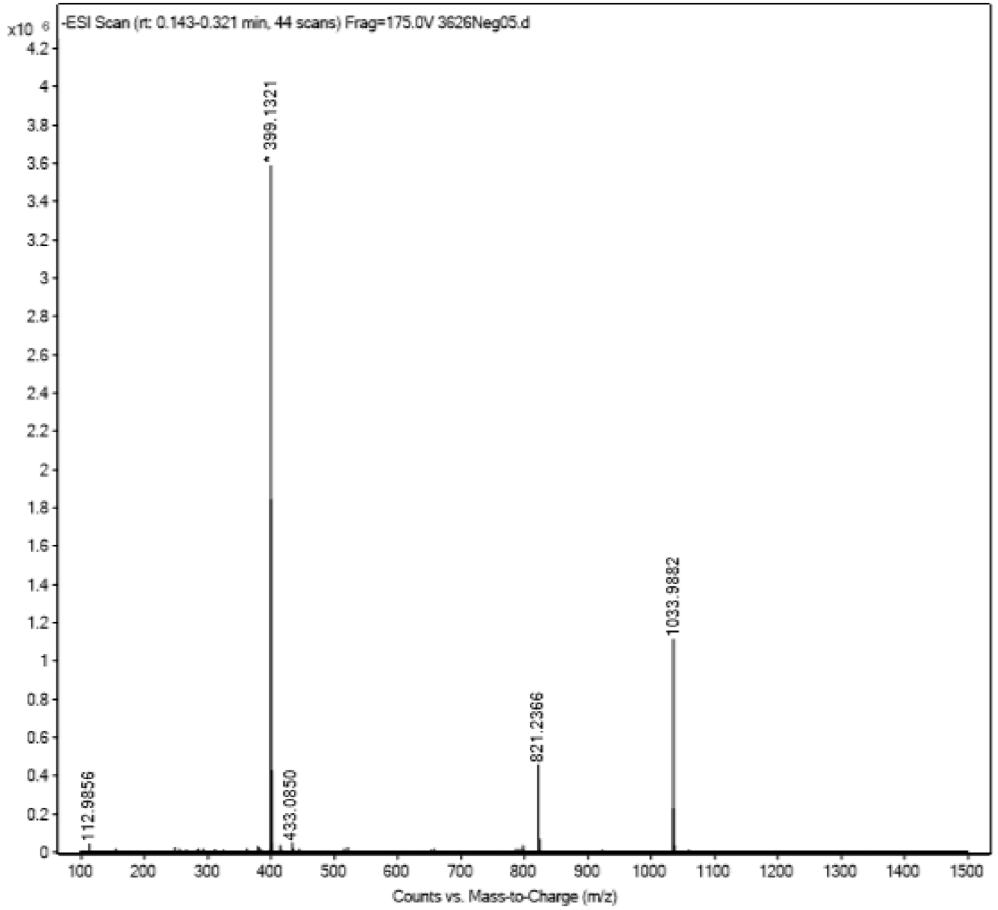

Figure S3. ESI-Mass spectrum from LC-MS/MS of interruptin A

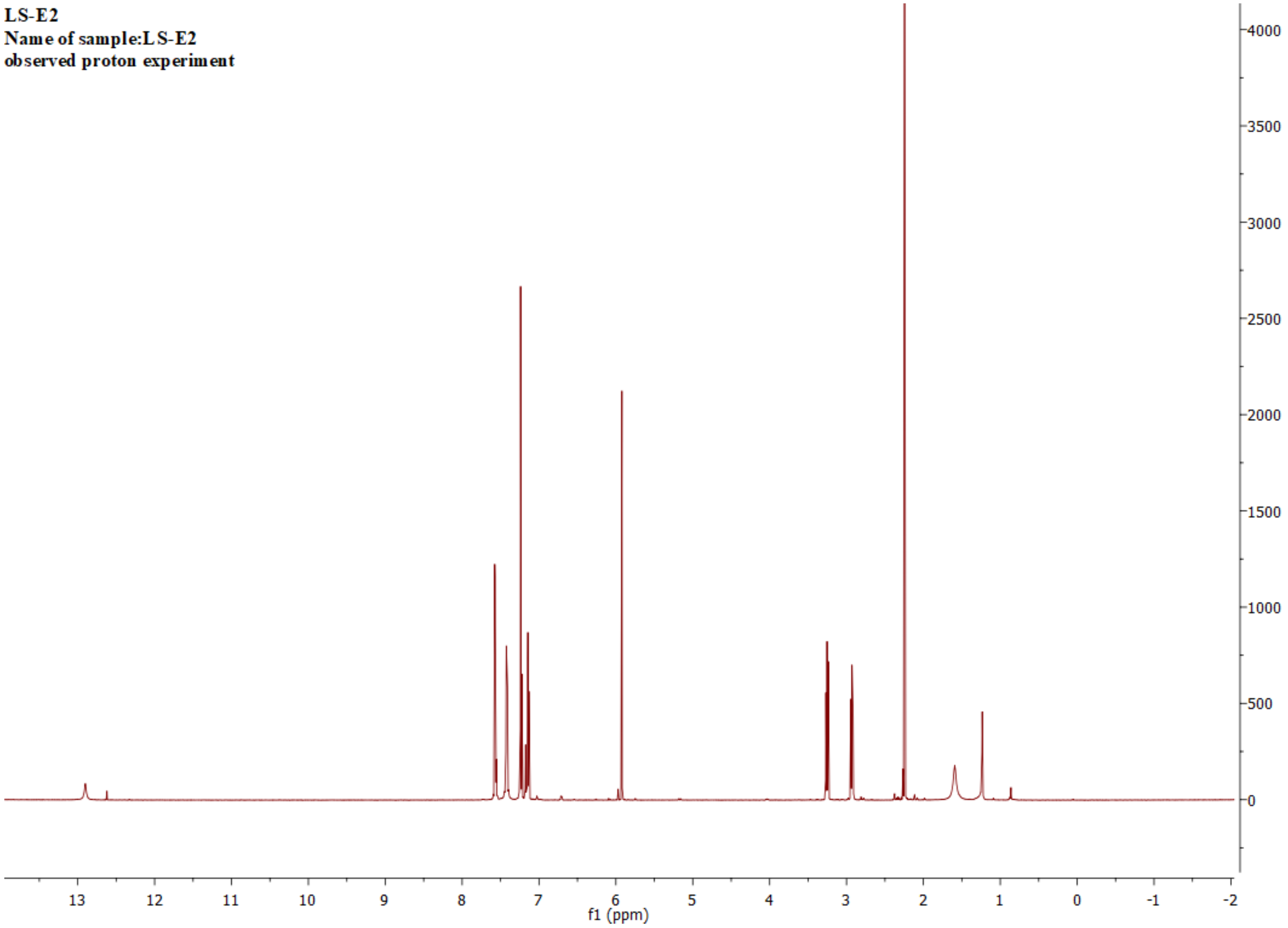

Figure S4. ^{1}H-NMR of interruptin A (500 MHz in $CHCl_3$-*d*)

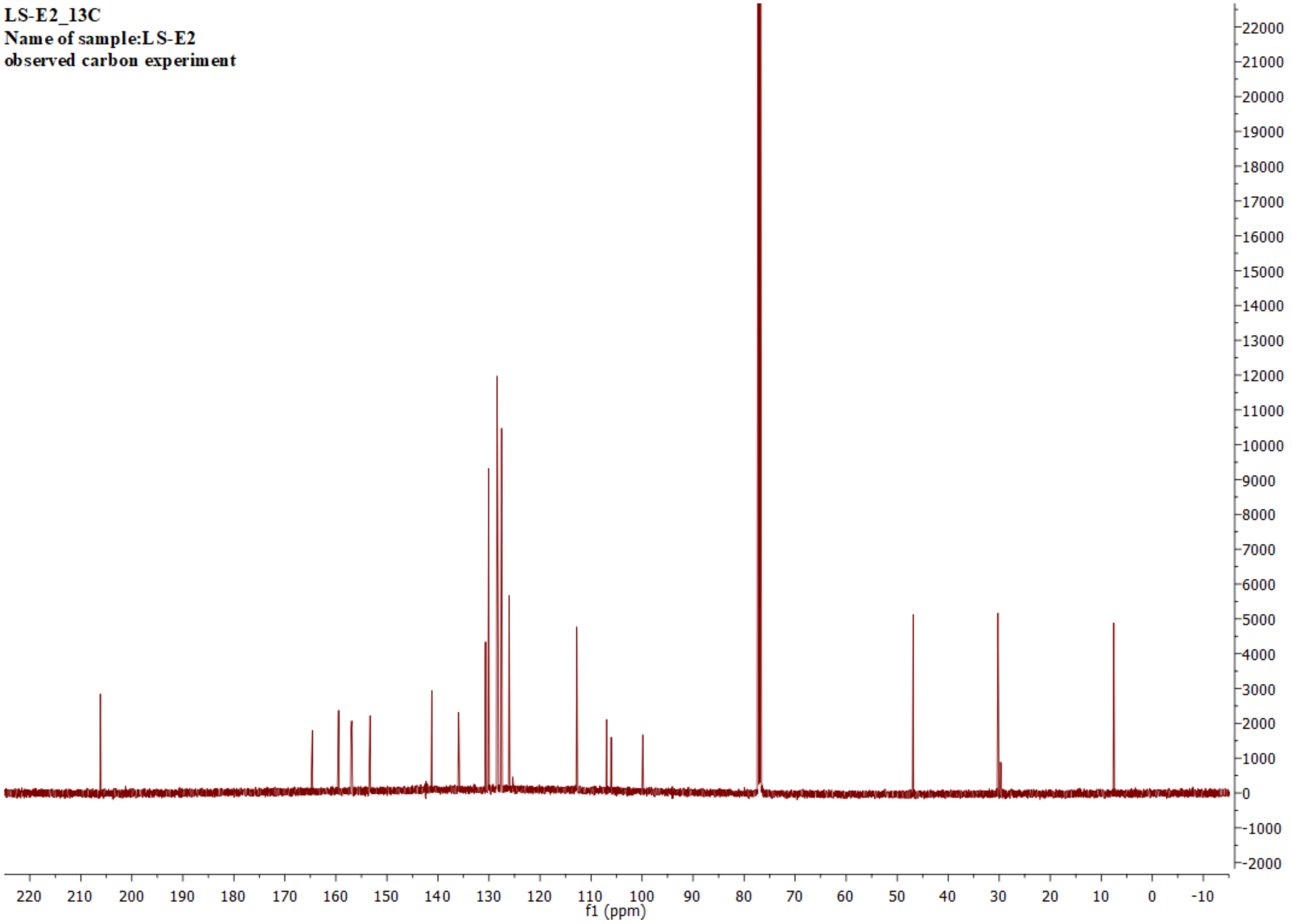

Figure S5. ^{13}C-NMR of interruptin A (125 MHz in $CHCl_3$-*d*)

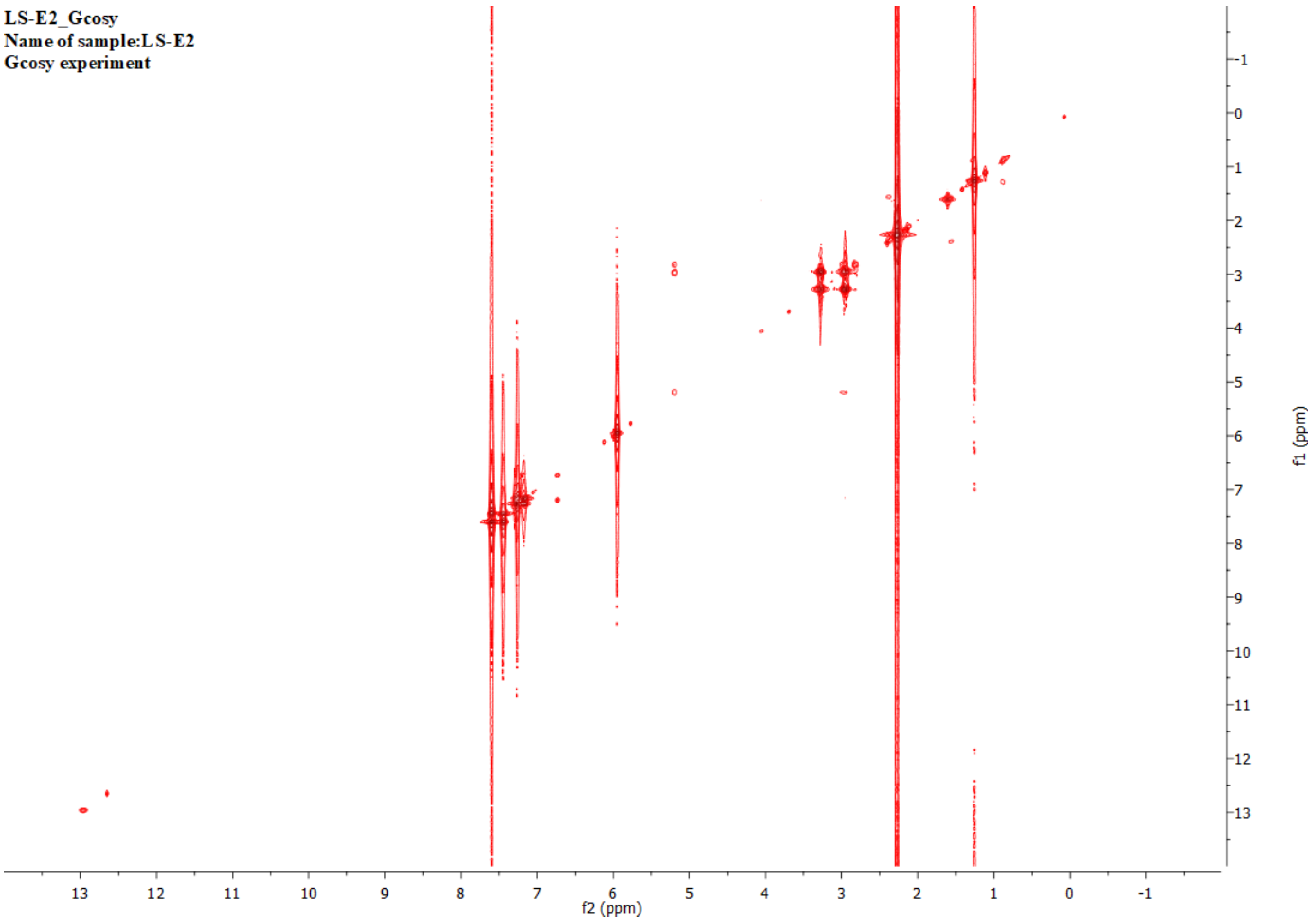

Figure S6. ^{1}H-^{1}H COSY of interruptin A

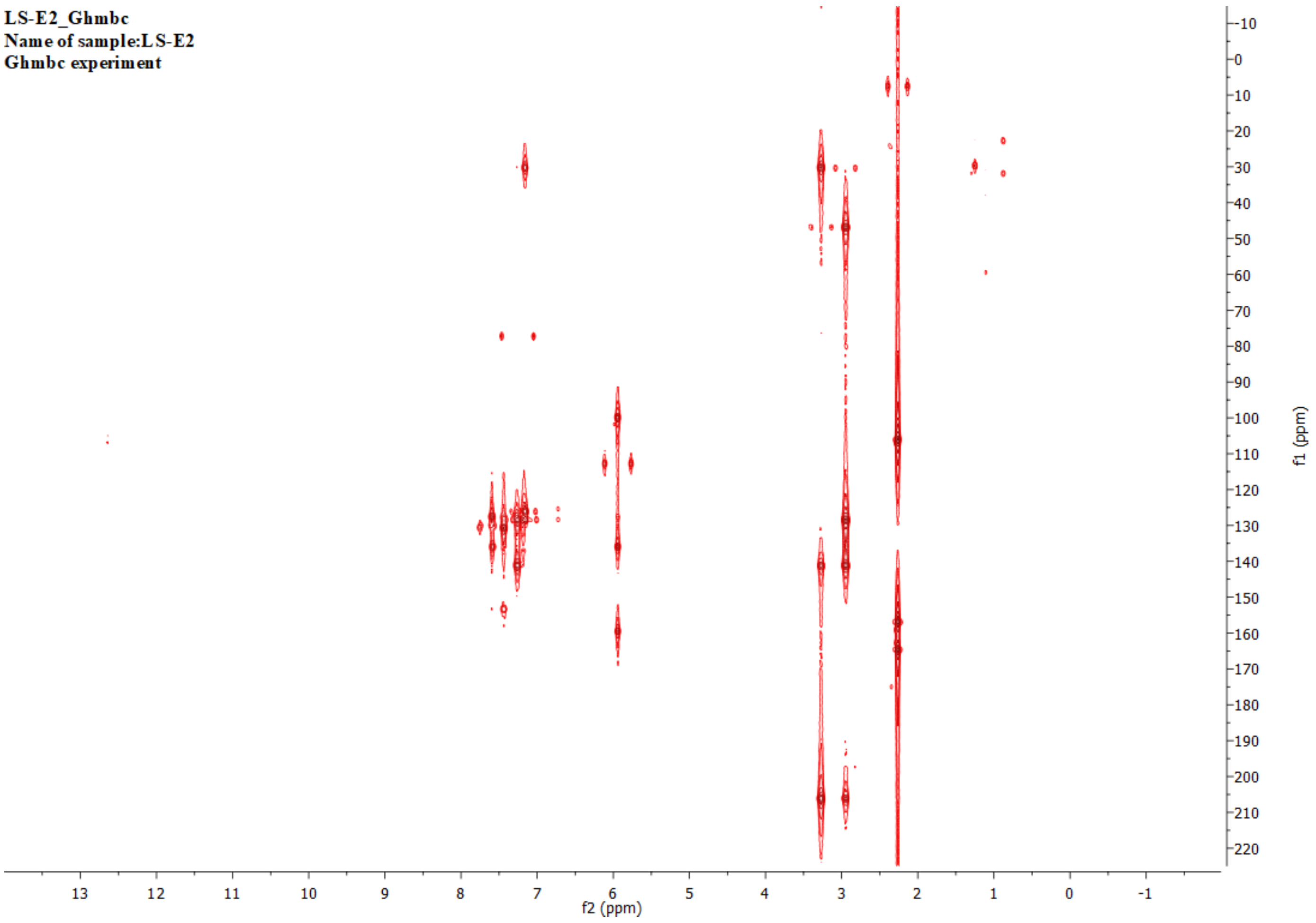

Figure S7. HMBC of interruptin A

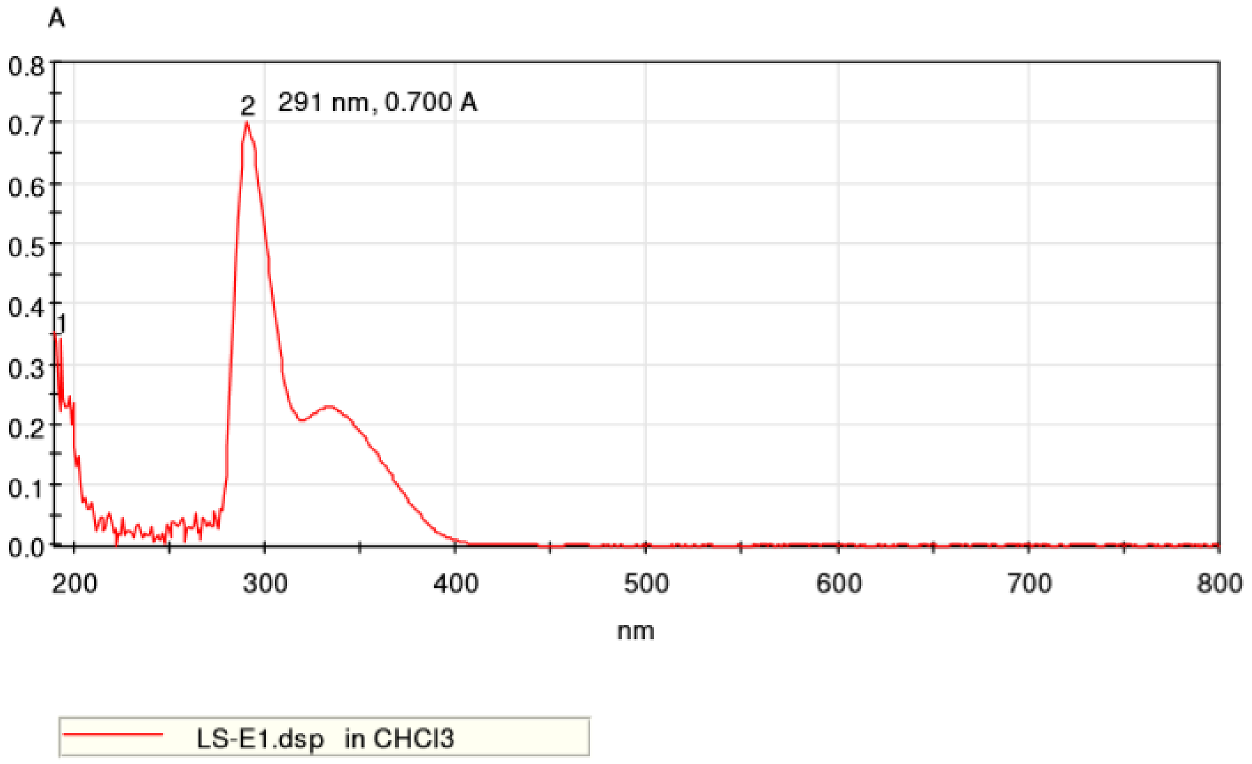

Figure S8. UV-Visible spectrum of interruptin C in chloroform

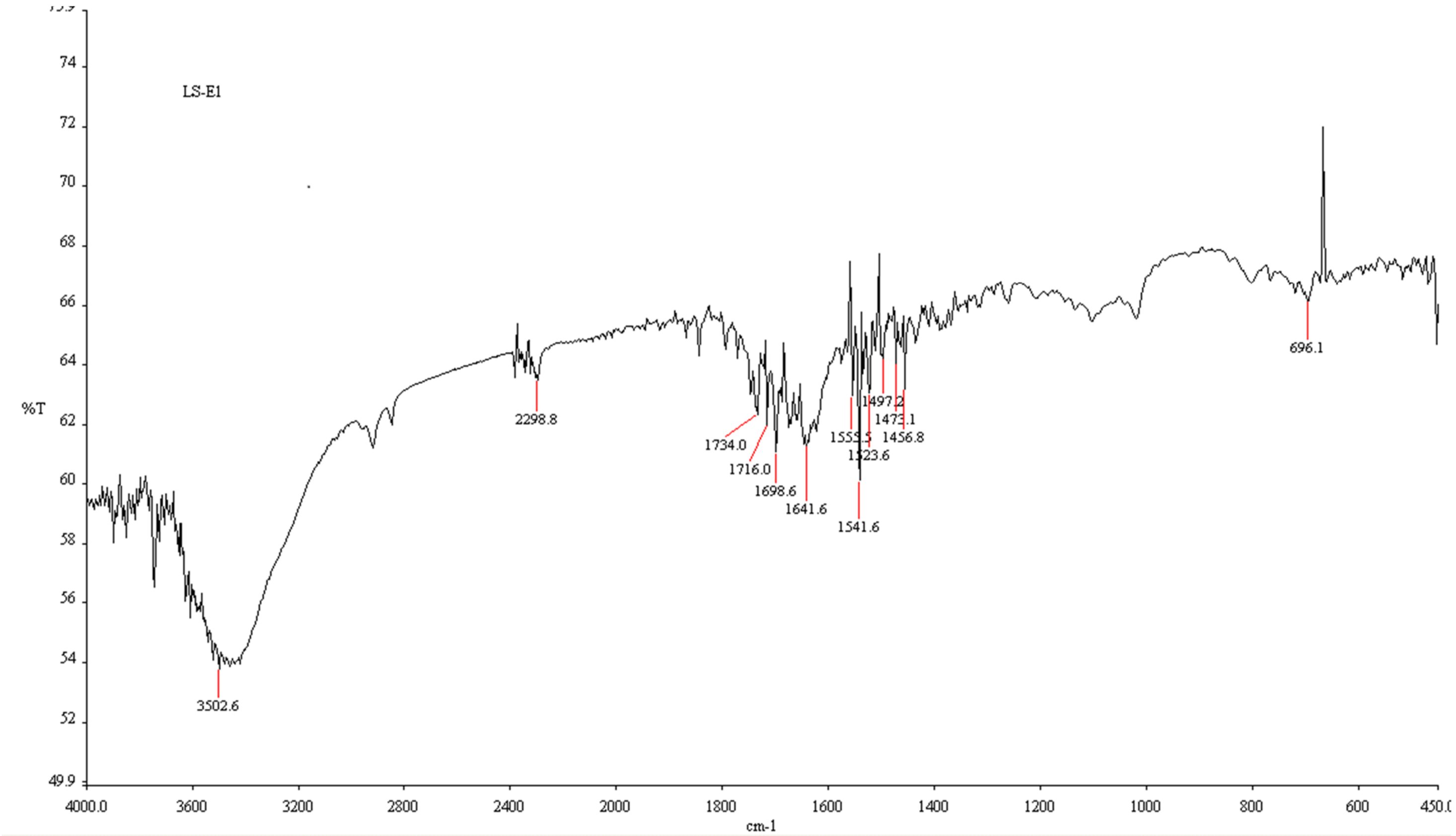

Figure S9. IR spectrum of interruptin C in KBr disc

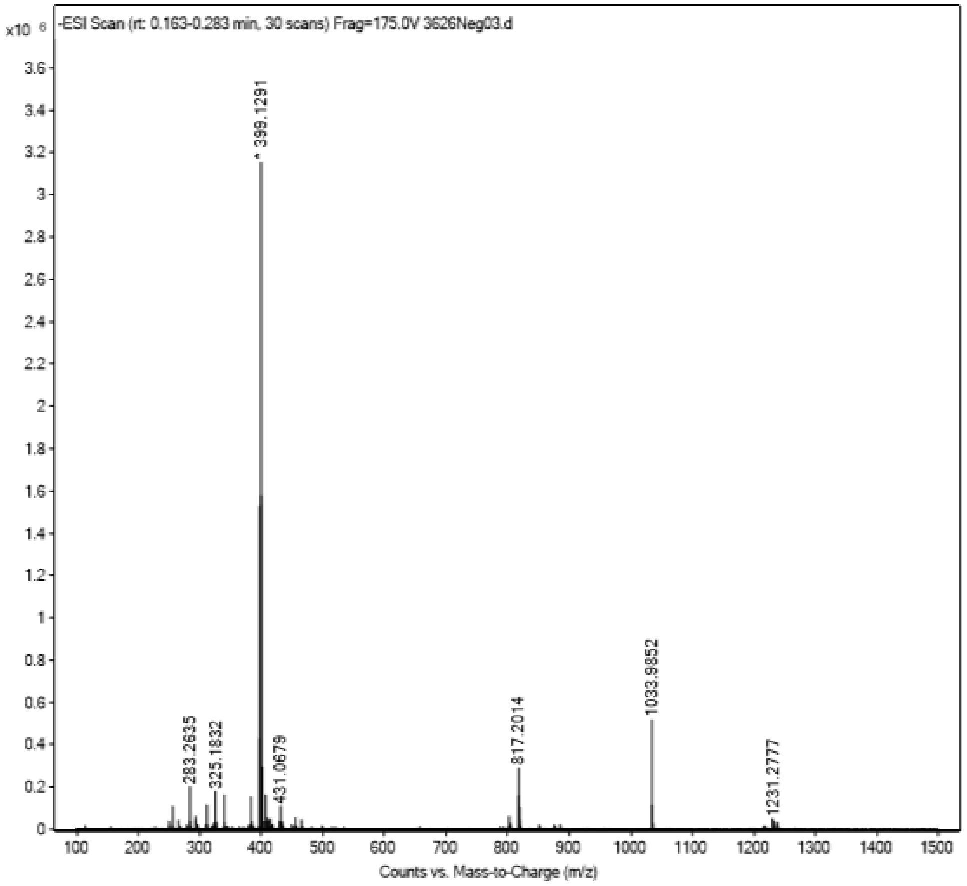

Figure S10. ESI-Mass spectrum from LC-MS/MS of interruptin C

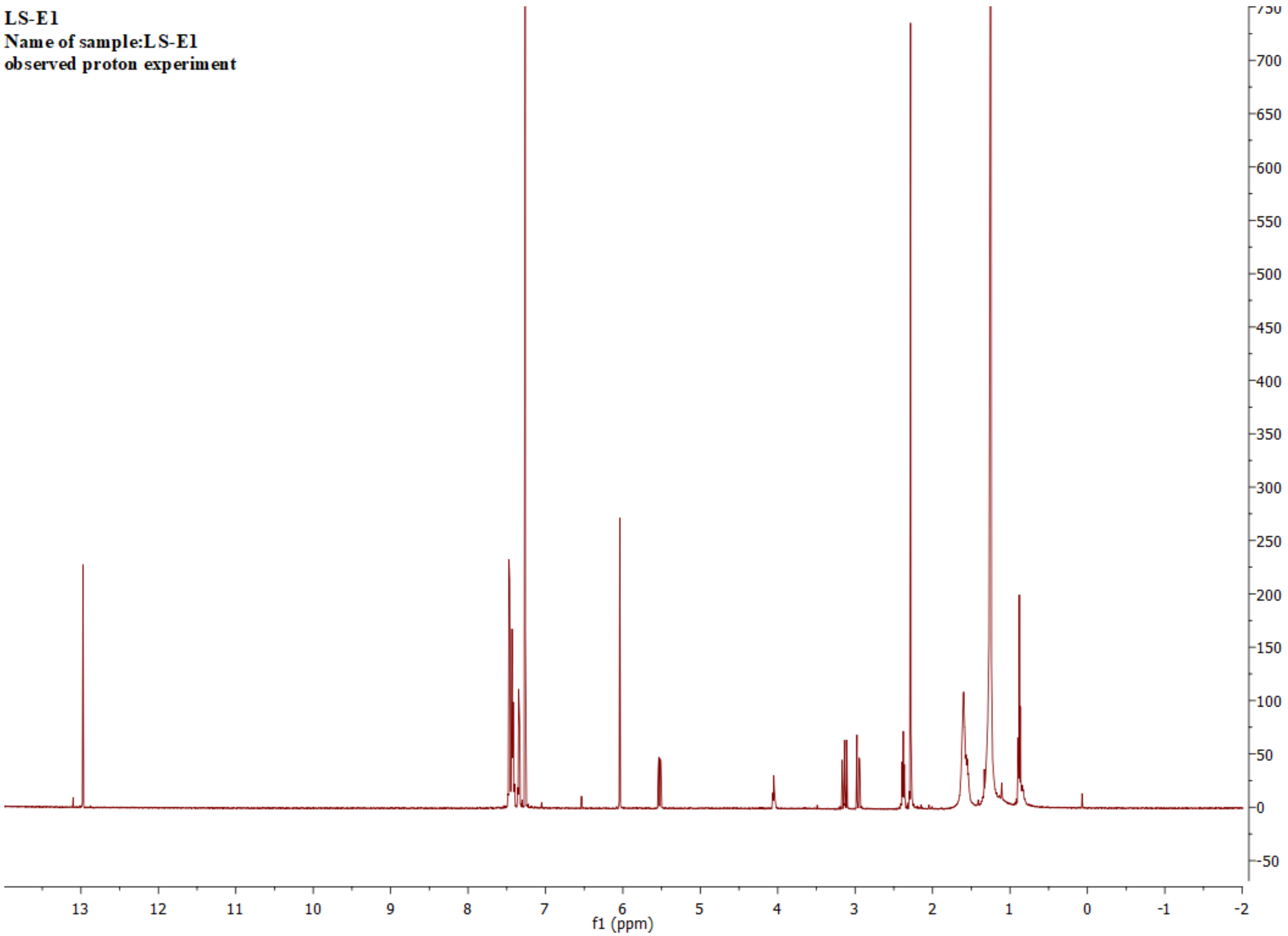

Figure S11. ^{1}H-NMR of interruptin C (500 MHz in $CHCl_3$-*d*)

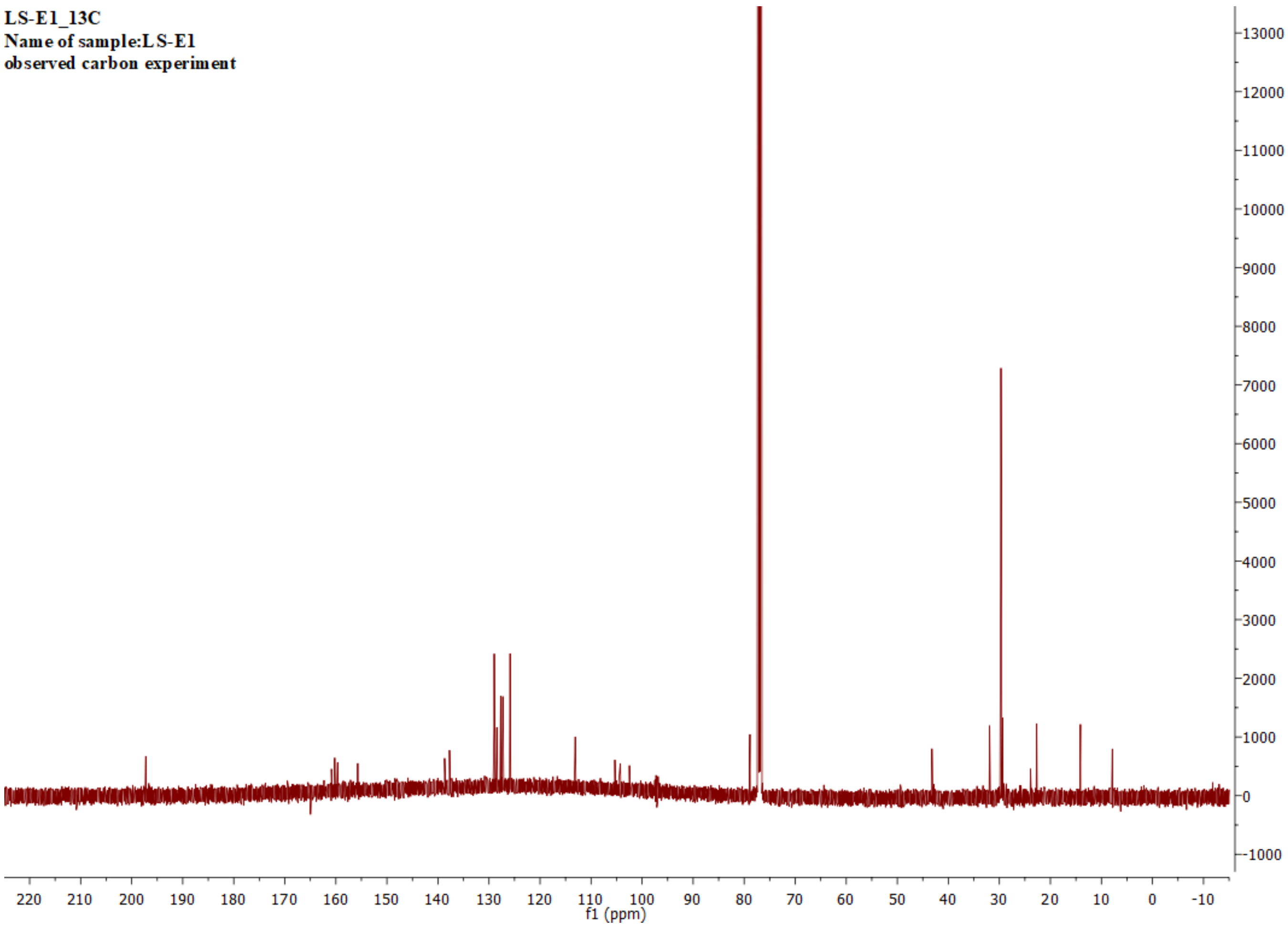

Figure S12. ^{13}C-NMR of interruptin C (125 MHz in $CHCl_3$-*d*)

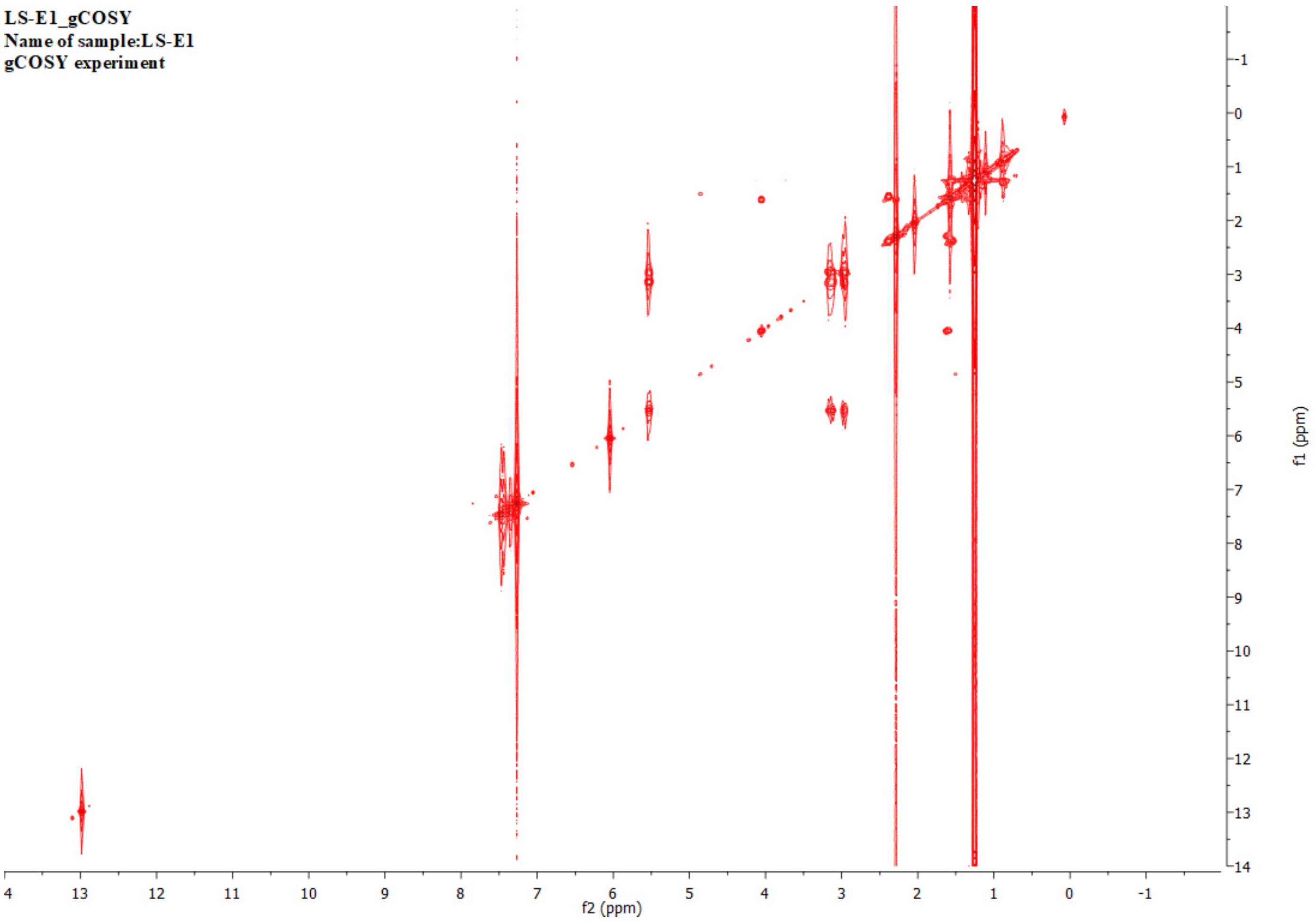

Figure S13. ^1H-^1H COSY of interruptin C

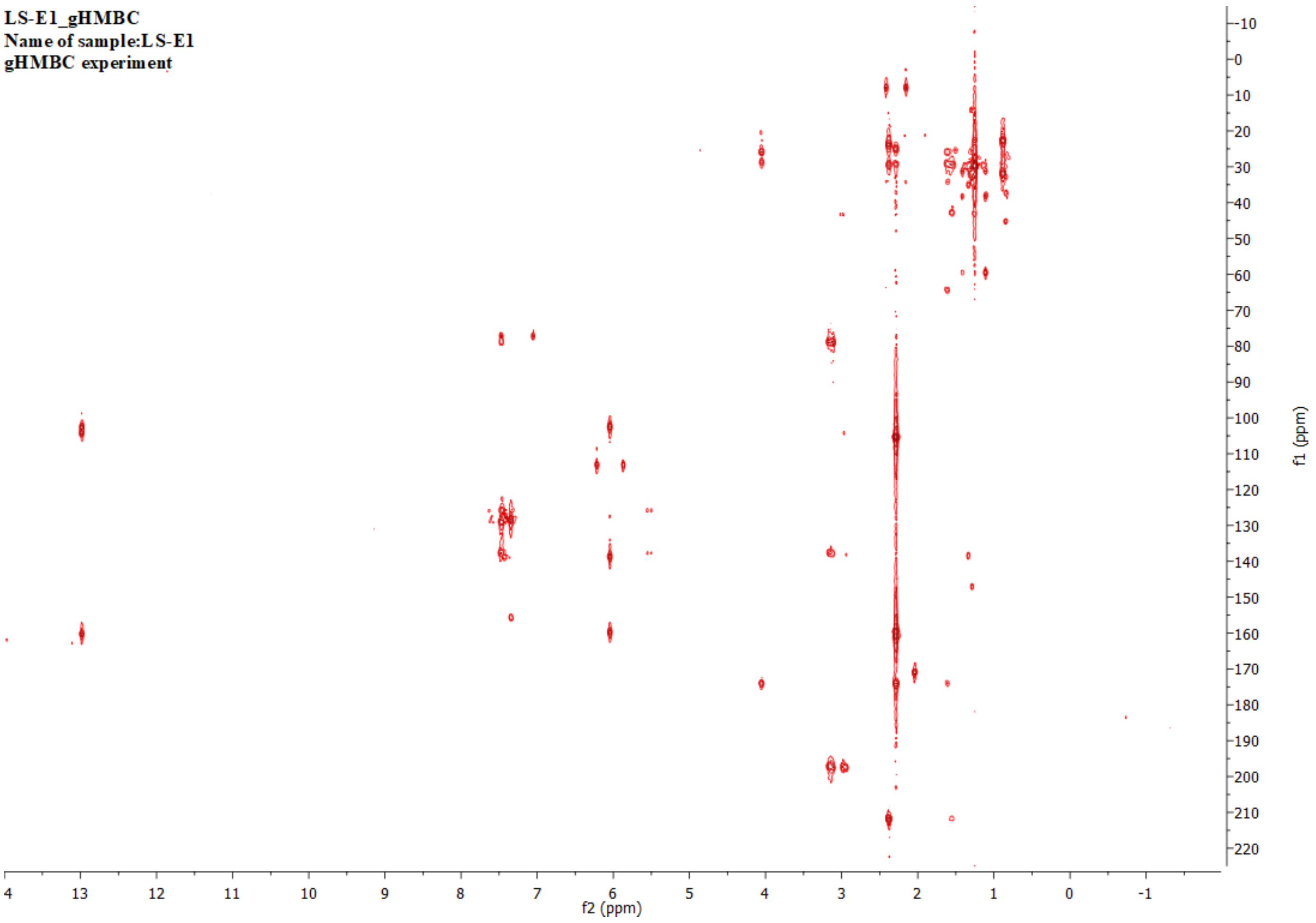

Figure S14. HMBC of interruptin C

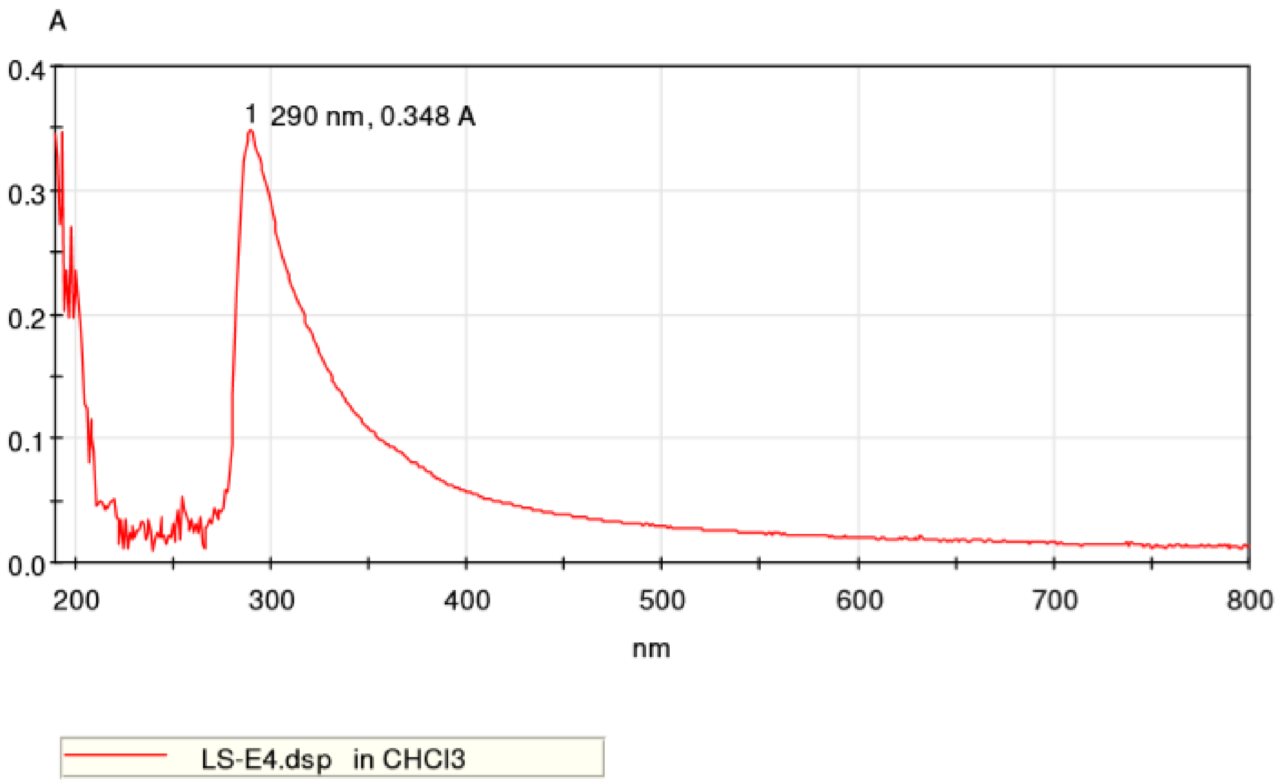

Figure S15. UV-Visible spectrum of ergosterol in chloroform

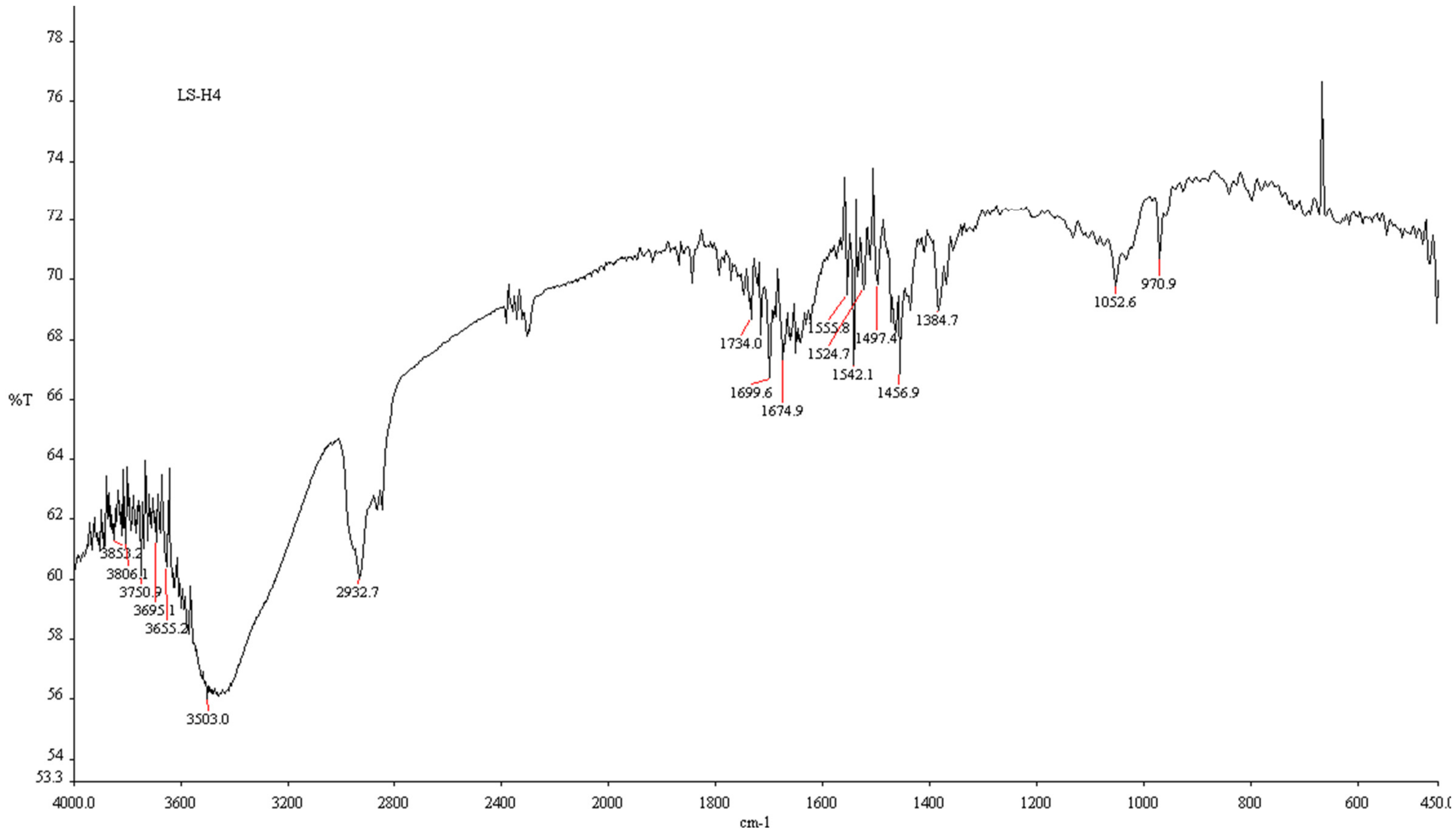

Figure S16. IR spectrum of ergosterol in KBr disc

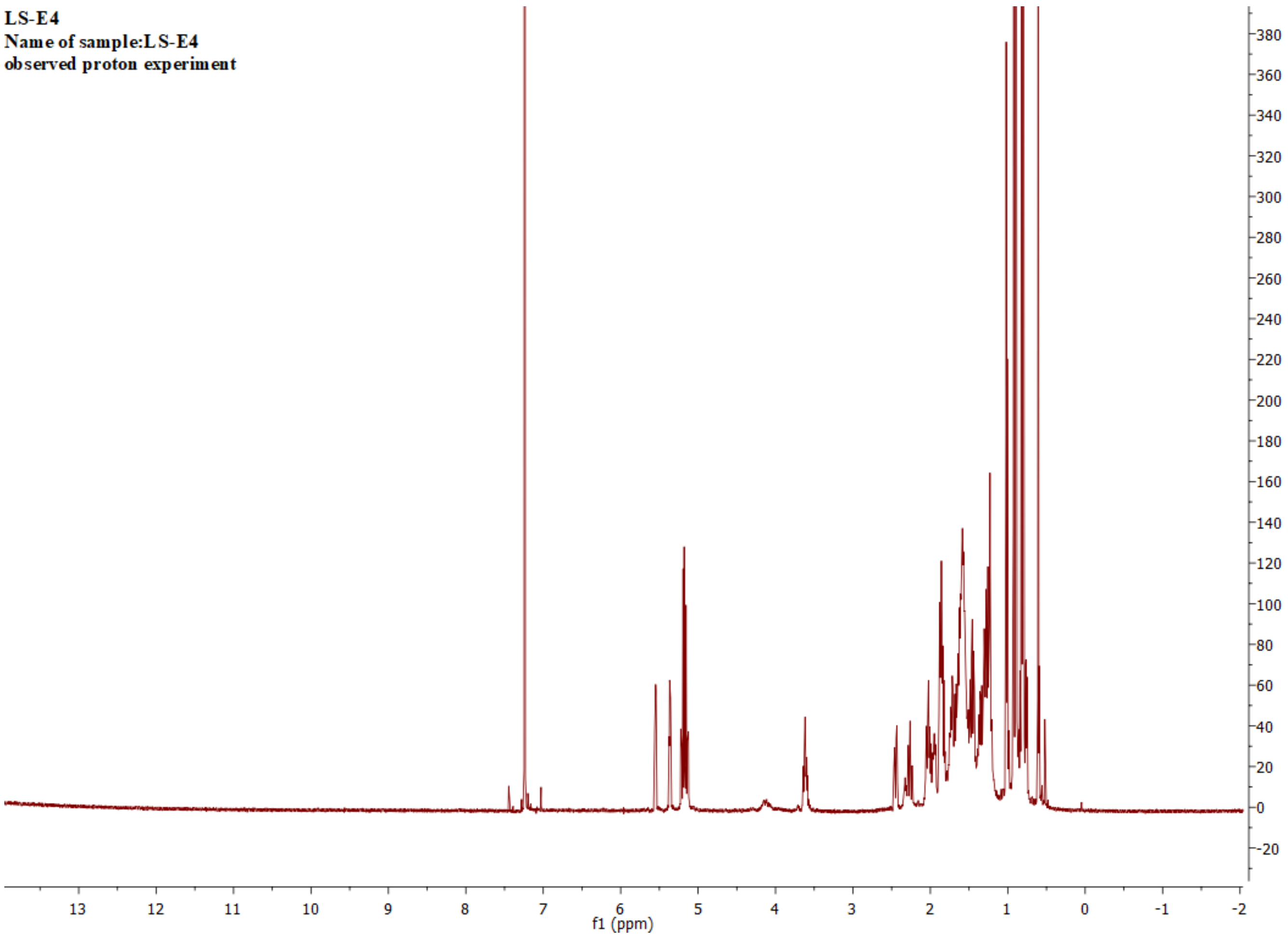

Figure S17. ^{1}H-NMR of ergosterol (500 MHz in $CHCl_3$-*d*)

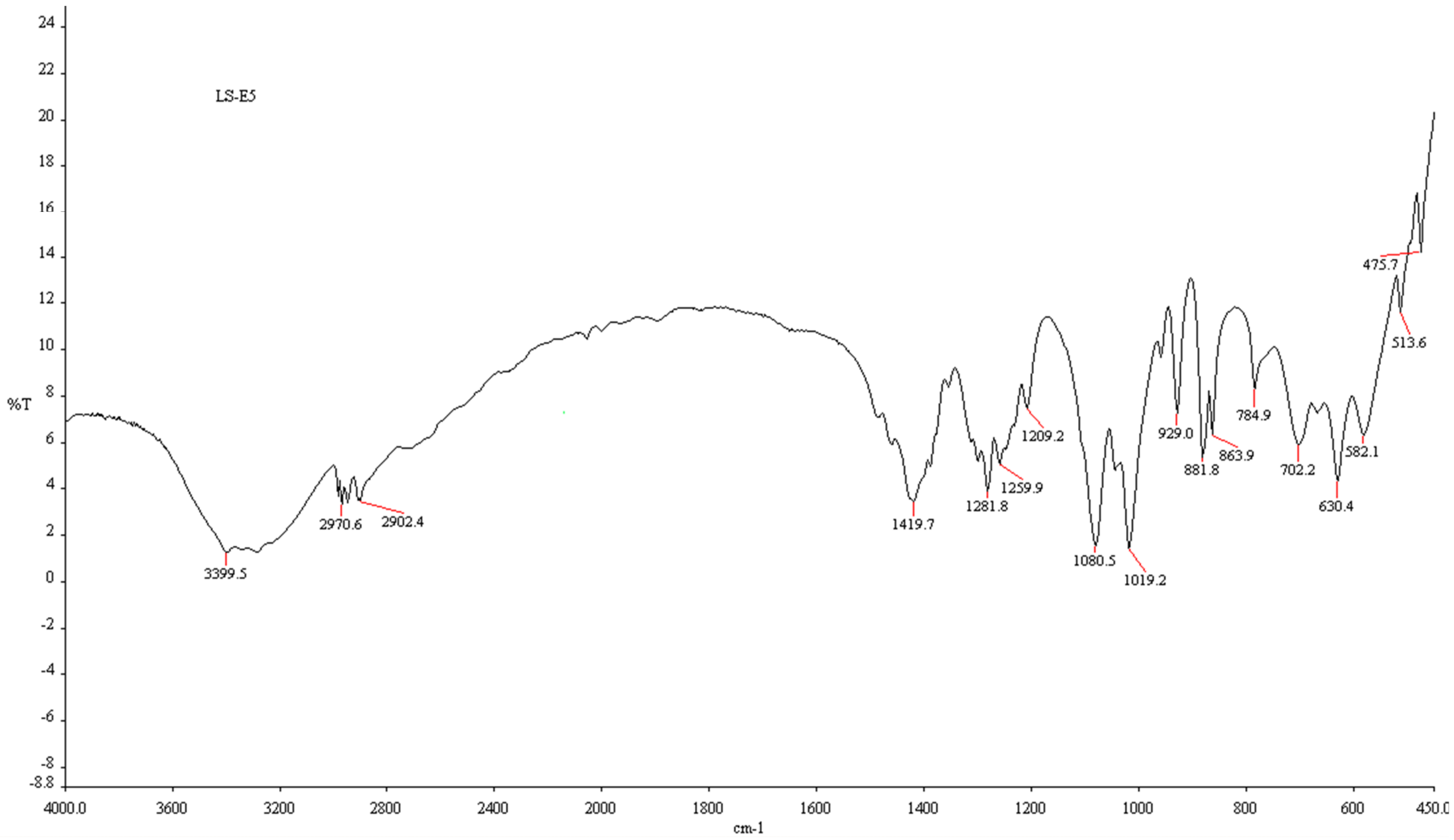

Figure S18. IR spectrum of diglycerol in KBr disc

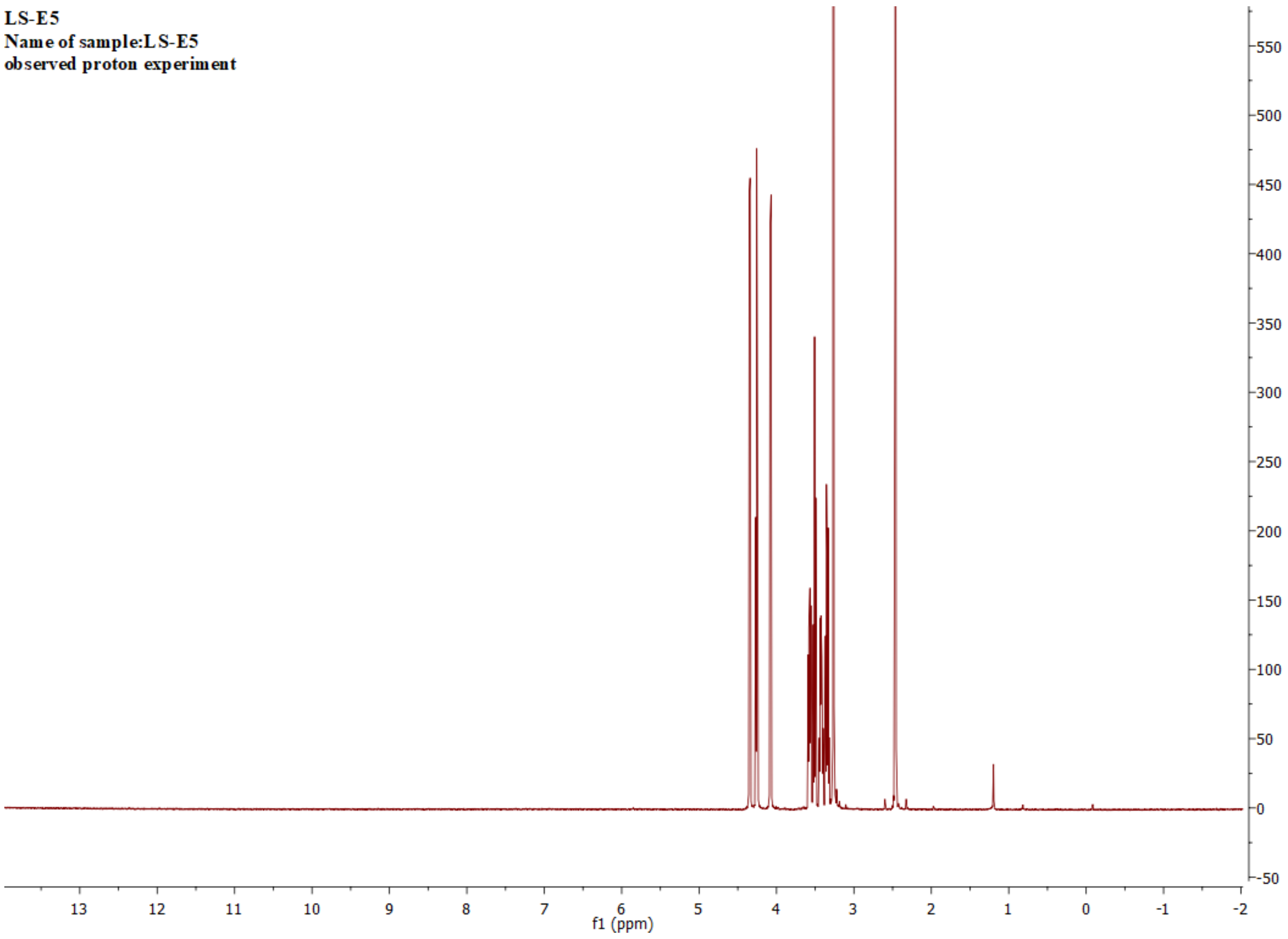

Figure S19. ^{1}H-NMR of diglycerol (500 MHz in DMSO-d_6)

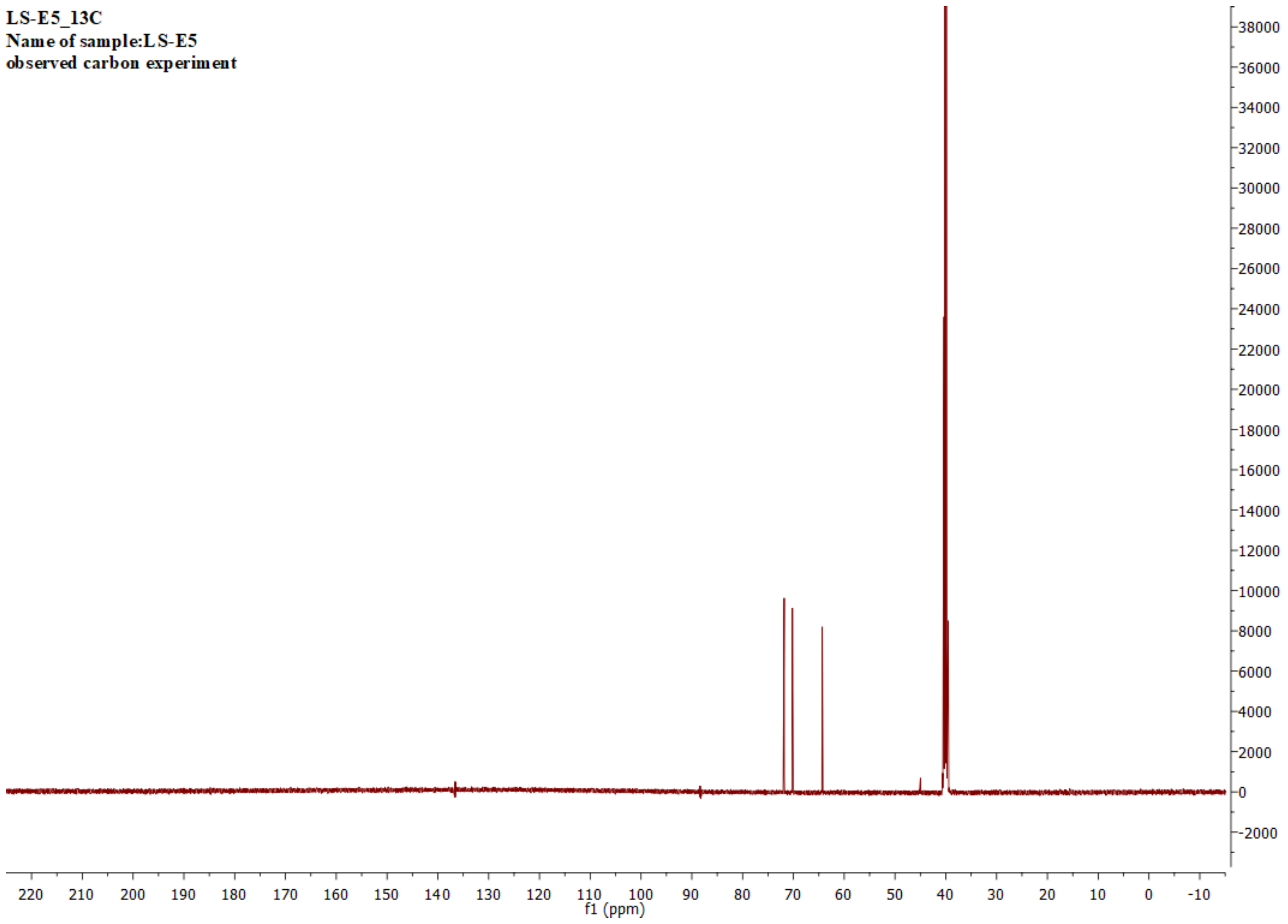

Figure S20. ^{13}C-NMR of diglycerol (125 MHz in DMSO-d_6)

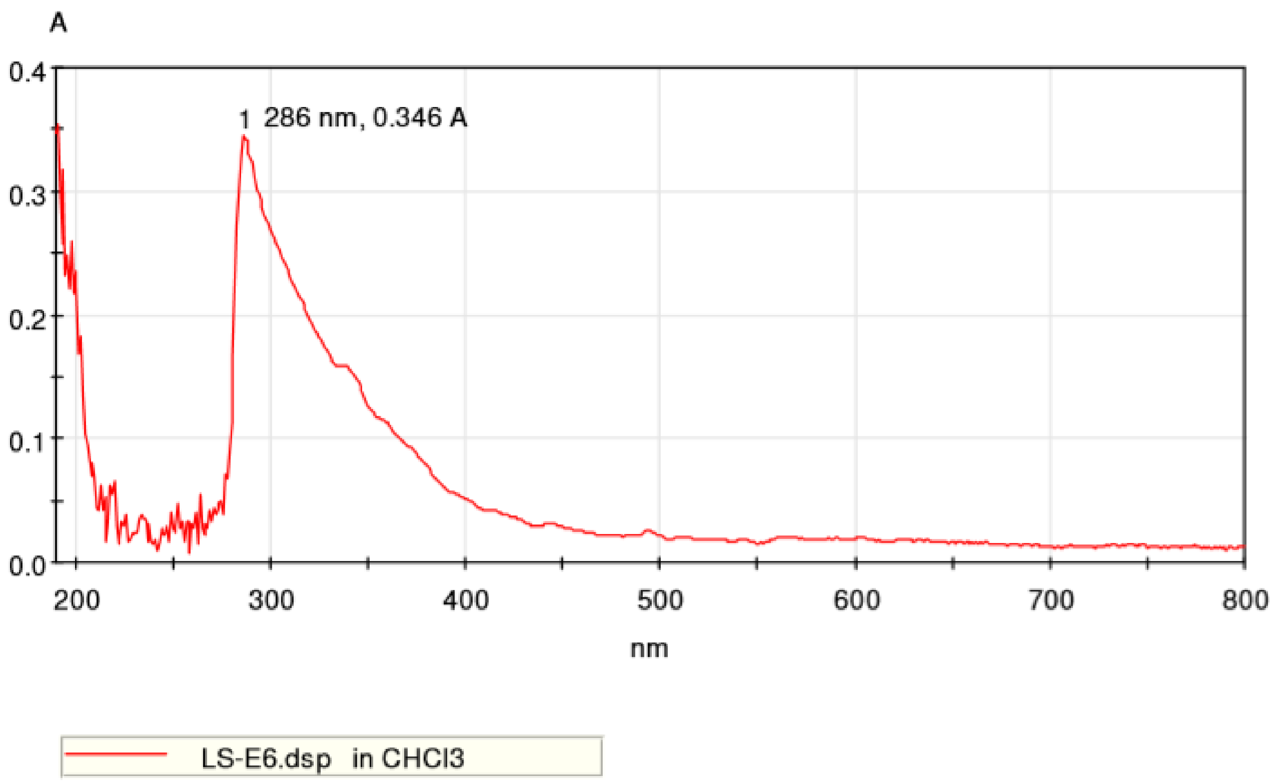

Figure S21. UV-Visible spectrum of the new diterpene in chloroform

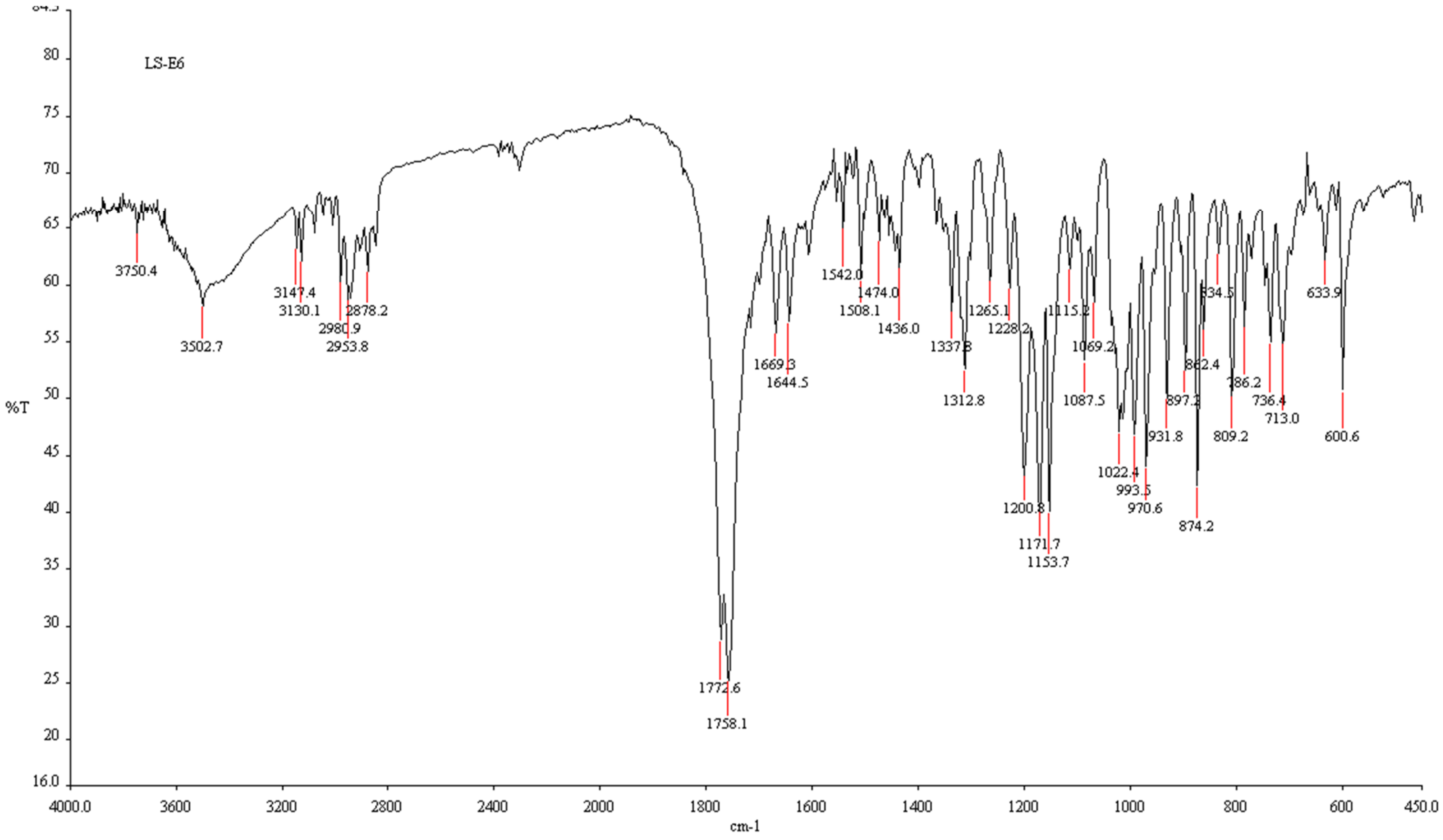

Figure S22. IR spectrum of the new diterpene in KBr disc

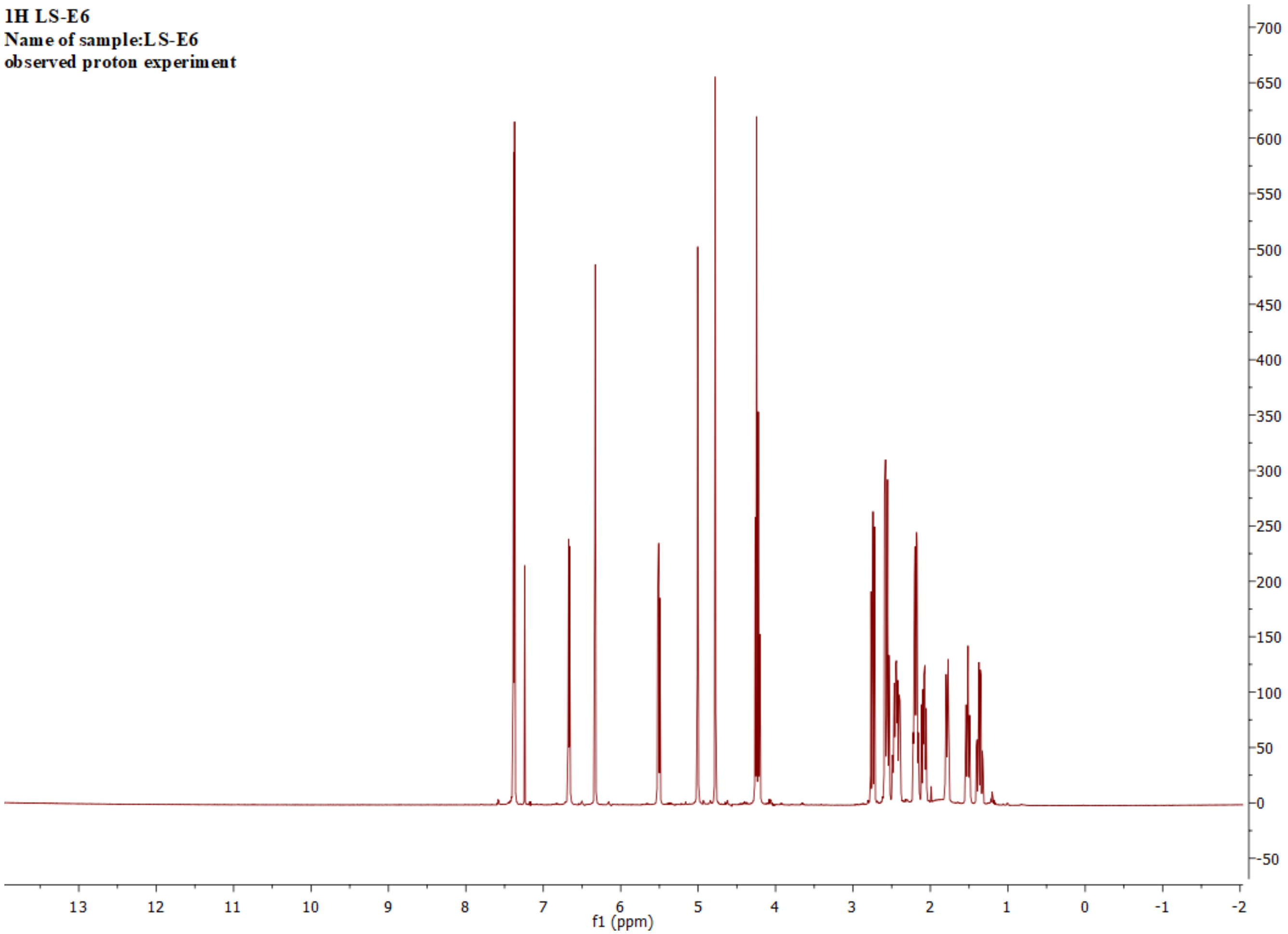

Figure S23. ^{1}H-NMR of the new diterpene (500 MHz in $CHCl_3$-*d*)

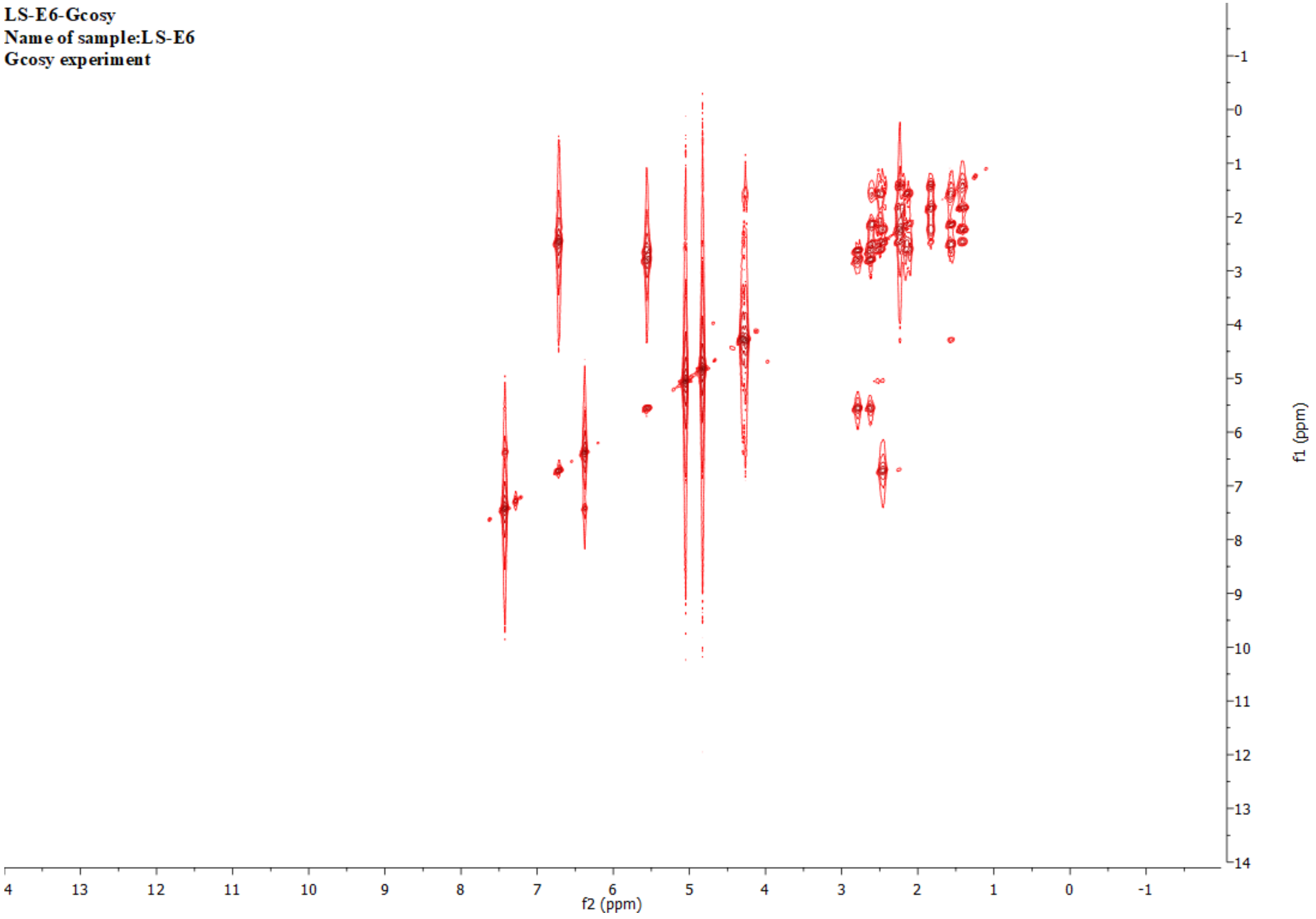

Figure S24. ^{1}H-^{1}H COSY of the new diterpene

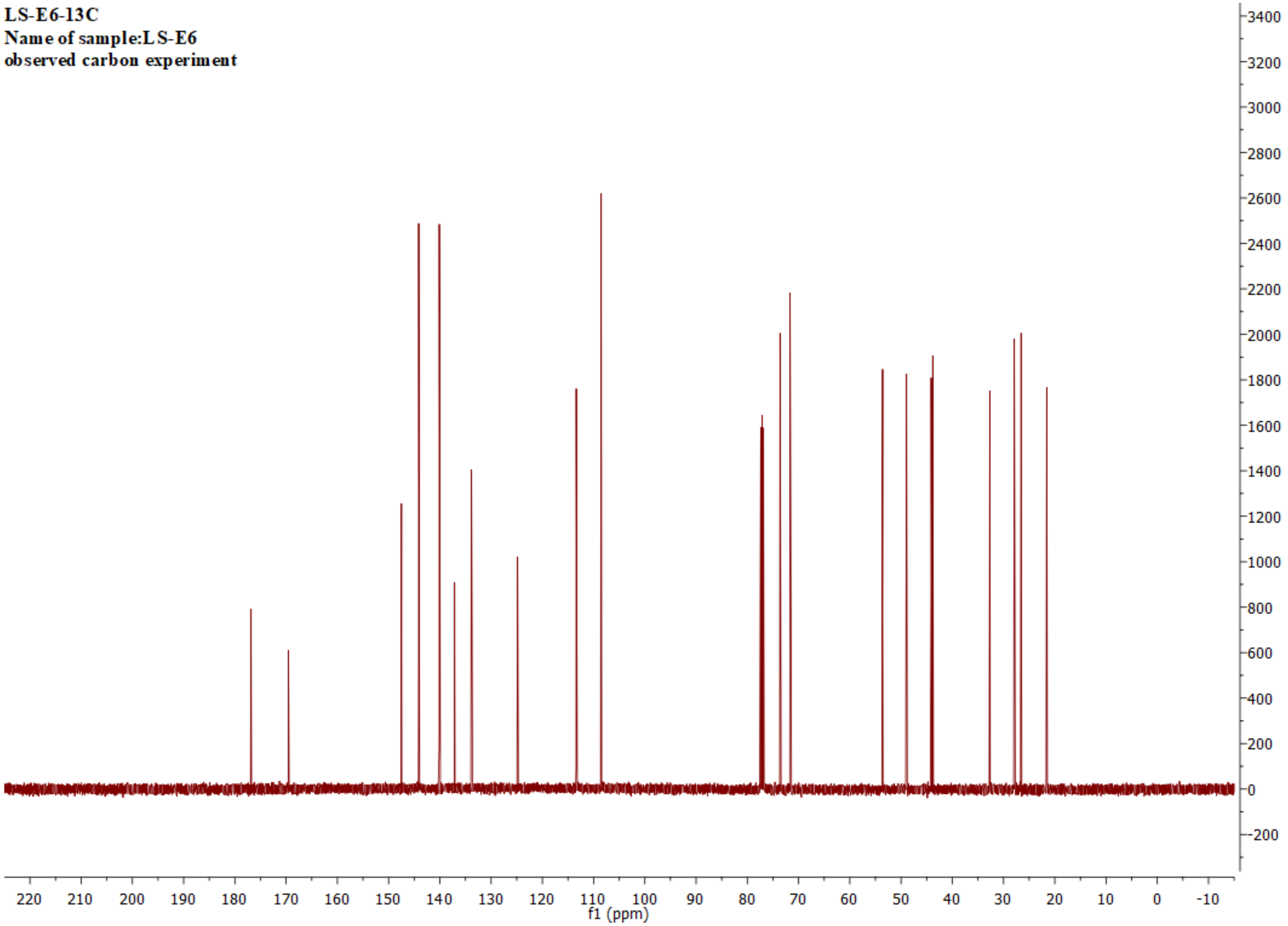

Figure S25. ^{13}C-NMR of the new diterpene (125 MHz in $CHCl_3$-*d*)

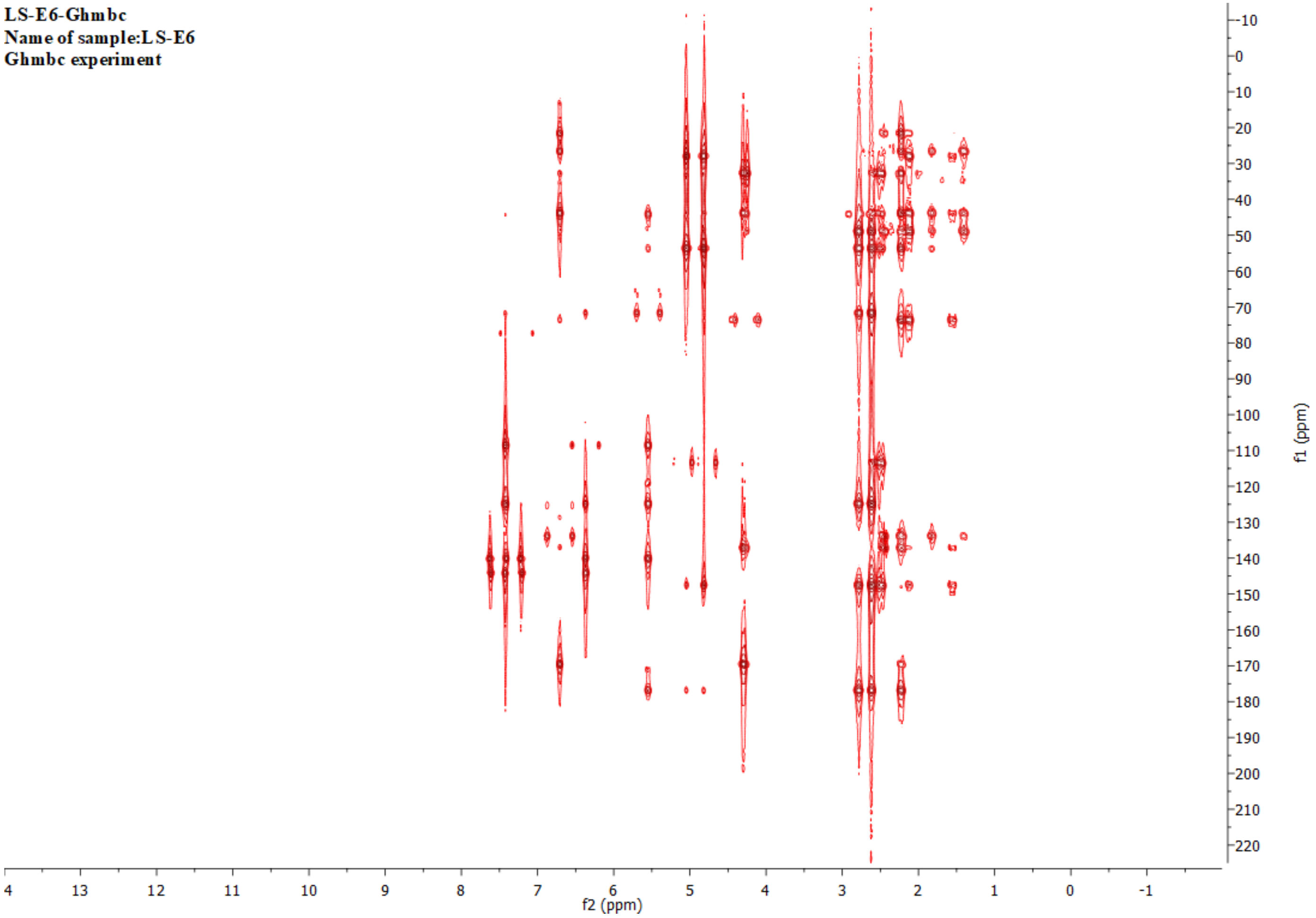

Figure S26. HMBC of the new diterpene

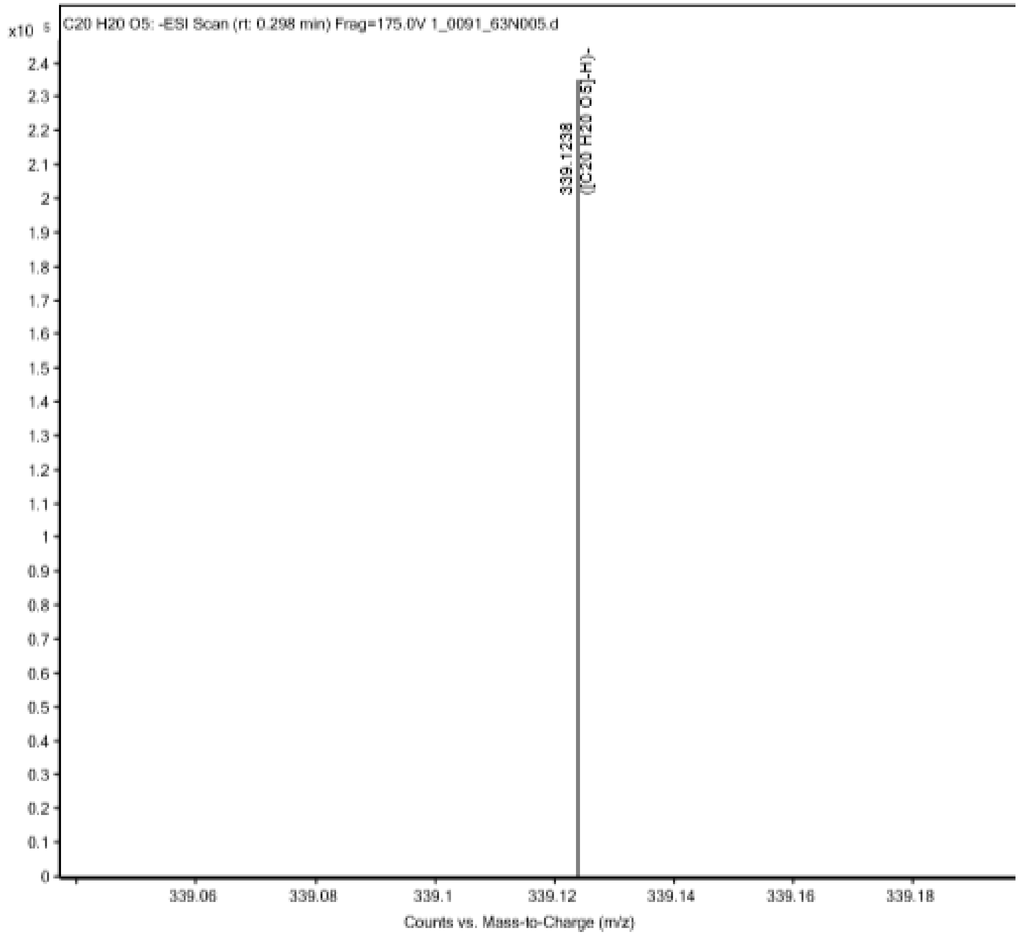

Figure S27. ESI-Mass spectrum from LC-MS/MS of the new diterpene

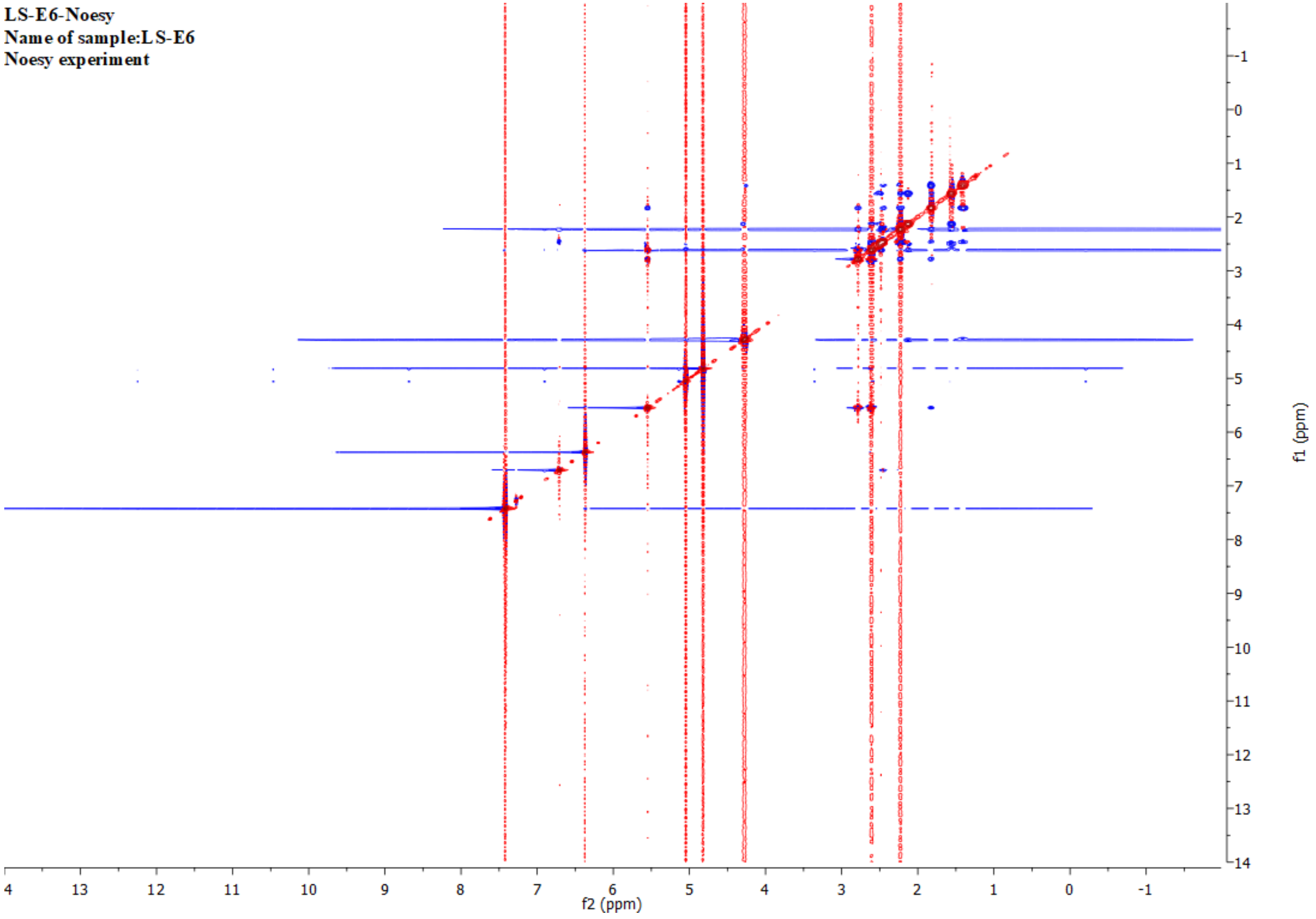

Figure S28. NOESY of the new diterpene
